# Lu_3_Al_5_O_12_:Ce^3+^ Fluorescent Ceramic with Deep Traps: Thermoluminescence and Photostimulable Luminescence Properties

**DOI:** 10.3390/ma18010063

**Published:** 2024-12-27

**Authors:** Junwei Zhang, Miao Zhao, Qiao Hu, Renjie Jiang, Hao Ruan, Hui Lin

**Affiliations:** 1 Engineering Research Center of Optical Instrument and System, Ministry of Education and Shanghai Key Lab of Modern Optical System, University of Shanghai for Science and Technology, No. 516 Jungong Road, Shanghai 200093, China; junweizhang@siom.ac.cn (J.Z.); linh8112@163.com (H.L.); 2 Aerospace Laser Technology and System Department, Wangzhijiang Innovation Center for Laser, Shanghai Institute of Optics and Fine Mechanics, Chinese Academy of Sciences, Shanghai 201800, China; miaozhao@siom.ac.cn (M.Z.); huqiao@siom.ac.cn (Q.H.); jiangrj@shanghaitech.edu.cn (R.J.)

**Keywords:** Lu_3_Al_5_O_12_, electron-trapping materials, optical data storage, photostimulable luminescence

## Abstract

Electron-trapping materials have attracted a lot of attention in the field of optical data storage. However, the lack of suitable trap levels has hindered its development and application in the field of optical data storage. Herein, Lu_3_Al_5_O_12_:Ce^3+^ fluorescent ceramics were developed as the optical storage medium, and high-temperature vacuum sintering induced the formation of deep traps (1.36 eV). The matrix based on the garnet-structured material ensures excellent rewritability. By analyzing the thermoluminescence and photostimulable luminescence, it is found that the transition of electrons provided by Ce^3+^ between the conduction band and trap levels offers the possibility for optical data storage. As evidence of its application, the optical information encoding using 254 nm light and decoding using a light stimulus and thermal stimulus were applied. These findings are expected to provide candidate material for novel optical storage technology, and further promote the development of advanced information storage technology.

## 1. Introduction

With the rapid development of society, the amount of global data increase exponentially. The huge amount of data puts forward challenges for information storage technology. Optical data storage (ODS) is widely used in information storage because of its outstanding performance, environmentally friendly nature, high capacity, and long lifetime [1,2,3]. However, caused by the limitation of optical diffraction resolution, the application of traditional ODS technologies is facing considerable challenges. The emergence of super-resolution recording [4] and information multiplexing technologies is expected to solve these problems [5,6,7,8,9]. To better apply the two technologies, a new optical storage medium needs to be developed.

Since Lindmayer first proposed the concept of electron-trapping materials (ETMs) in 1986 [10], they have been widely used in data storage [11,12,13], emergency display [14], and information processing [15]. ETMs, which can retain excitation energy and release it as light emission upon external thermal and optical stimulations, are also referred to as photostimulable luminescence (PSL) materials. PSL materials have great application prospects in the field of ODS due to its all-optical operation mode [16]. Some sulfide-based PSL materials have been developed in the previous period, but most of them focus on optical computing operations [17,18,19,20]; there are few studies on ODS performance, and the problems of sulfide pollution and poor thermal stability limit its applications. The emergence of ceramic phosphors solves the above problems and exhibits good ODS properties, such as rewritability and high speed of reading/writing. Some researchers have prepared organic–inorganic composite fluorescent films. Zhuang et al. proposed two PSL materials (Sr_1-x_Ba_x_)Si_2_O_2_N_2_:Eu/Yb, Dy and NaMgF_3_:Tb that can complete the writing and reading of information by 405 nm and 980 nm laser/heating, which allows for information multiplexing; Deng et al. prepared Y_2_GeO_5_:Pr, Tb PSL materials that can record information in a bit by bit mode by a 515 nm laser, which demonstrated the potential of PSL materials in the field of high-density ODS [21,22,23]; Lin et al. prepared phosphor-in-glass BaSi_2_O_5_:Eu, Nd for data storage, and established the relationship between trap and PSL performance [24]. As a result of the poor stability and uniformity of these materials, they are not ideal materials for data storage. Cerium-doped lutetium aluminum garnet (Lu_3_Al_5_O_12_:Ce) ceramic is a promising ceramic material with excellent optical and thermal properties, good chemical stability, and high mechanical strength, which has been widely used in the fields of fluorescent materials, laser materials, and scintillation materials [25,26,27,28,29]. The above characteristics are very suitable as optical storage matrix materials.

However, the lack of appropriate depth of traps limits the application of new information storage materials. The trap levels of ETMs should be considered. Generally, the trap depth is between 1 and 1.5 eV, which can not only ensure the stable storage of information, but also be read out by near-infrared light [24,30]. Ion doping is a typical modulation method to construct traps. Xie et al. reported a series of Y_3_Al_5-x_Ga_x_O_12_:Ce^3+^, V^3+^ PSL materials with a tunable trap depth (1.2–1.6 eV). According to the vacuum referred binding energy (VRBE) diagram, the effect of Ga^3+^ doping on the formation of deep traps was analyzed [31]. In the typical spinel-structured system, there are lots of anti-site defects of Ga^3+^ and Mg^2+^, and the doping of Bi^3+^/Cr^3+^ can induce the formation of deep traps [32,33].

In this work, Lu_3_Al_5_O_12_:Ce^3+^ PSL materials were developed by vacuum sintering technology. Its spectral properties and crystal structure were studied in detail, capable of emitting 512 nm green light, and SEM shows that the grain size distribution is between 10 and 12 μm. In particular, the origin of the trap and the mechanism of optical storage were analyzed by thermoluminescence curves. Then, its rewritability and information retention ability were tested. It also exhibited the writing/reading process of the optical information by a 254 nm light and 980 nm laser/heating.

## 2. Experiments

Lu_3−x_Al_5_O_12_:xCe (x = 0.011, 0.013 and 0.015) (abbreviated as LuAG:xCe) materials were prepared by the vacuum sintering technique. The required chemical regents include Lu_2_O_3_ (4N), Al_2_O_3_ (4N), CeO_2_ (4N), MgO, and TEOs. Firstly, raw materials were mixed with anhydrous alcohol according to the stoichiometric ratio and ground in a planet-type ball mill at 230 rpm for 12 h. The ball-milled mixtures were rinsed with anhydrous alcohol and dried in an oven at 80 °C for 6 h. Then, the dried samples were thoroughly ground. After grinding, the dried powders were sieved through a 200-mesh screen. Prior to sintering, the green bodies were shaped from powders under a uniaxial press of 30 MPa and were cold isostatic pressed (CIP) at 210 MPa. The obtained samples were pressed and pre-sintered at 800 °C for 6 h and high-temperature sintered at 1850 °C for 6 h; the preparation flow chart of fluorescent ceramics as shown in Figure S1.

X-ray diffraction (XRD) patterns were obtained by an X-ray diffractometer, and the test range is 2θ = 10–90° (Bruker D8 ADVANCE, Karlsruhe, Germany). The steady photoluminescence (PL) and photoluminescence excitation (PLE) were tested by a fluorescence spectrophotometer (Hitachi F-4600, Tokyo, Japan). The morphologies of the ceramics were measured using a scanning electron microscope (SEM, Verios G4 UC, Waltham, MA, USA). Thermoluminescence (TL) was tested by a TOSL-3DS meter (Guangzhou Rongfan, Guangzhou, China). Luminescence decay curves and Photostimulated luminescence (PSL) spectra were measured by a Time-resolved spectrometer (Edinburgh Instruments, FLSP-920, Livingston, UK).

## 3. Results and Analysis

### 3.1. Structure and Optical Properties

**Figure 1:**
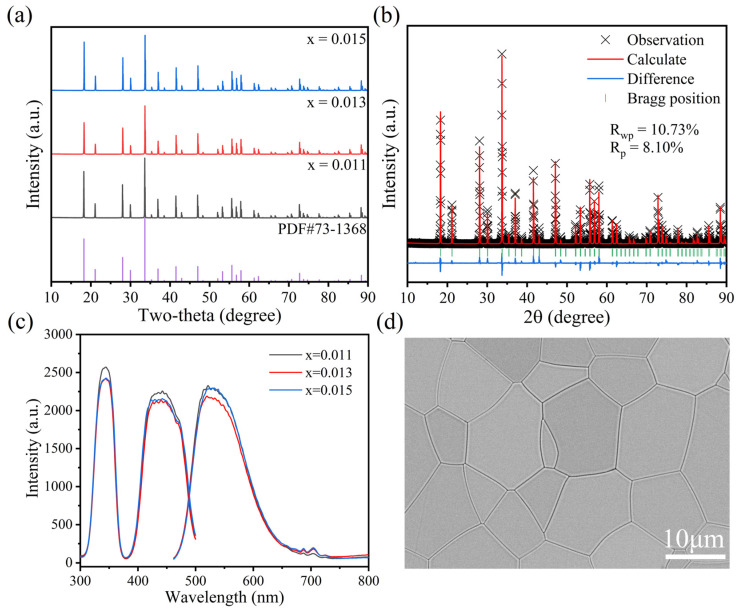
(**a**) XRD patterns of LuAG:xCe (x = 0.011, 0.013 and 0.015). (**b**) Rietveld refinement of LuAG:0.013Ce. (**c**) PLE and PL spectra of LuAG:xCe (λ_em_ = 512 nm, λ_ex_ = 437 nm). (**d**) SEM image of LuAG:0.013Ce^3+^.

As shown in Figure 1a, we tested the XRD spectrum of the Lu_3−x_Al_5_O_12_:xCe (x = 0.011, 0.013 and 0.015), and the diffraction peaks are in good agreement with the standard XRD pattern of Lu_3_Al_5_O_12_ (NO. 73-1368); the space group is Ia–3d (NO. 230), and no obvious impurity peaks are observed. This indicates that Ce^3+^ is doped into the Lu_3_Al_5_O_12_ lattice, forming a solid solution. Different concentrations of Ce^3+^ doping have little effect on the lattice structure of LuAG, and the peak positions are basically consistent [29,34]. The calculated lattice parameters are a = b = c =11.8903 Å, α = β = γ = 90°. The XRD Rietveld refinement of the Lu_2.987_Al_5_O_12_:0.013Ce^3+^ is presented in Figure 1b; the reliability parameters Rwp, Rp, and χ^2^ were calculated at 10.73%, 8.10%, and 10.04. Almost all the peaks are well-indexed with the Lu_3_Al_5_O_12_ material, which indicates the results are reliable. Figure 1c presents the PLE (λ_em_ = 512 nm) and PL (λ_ex_ = 437 nm) of the LuAG:xCe^3+^ material. The excitation spectrums have two peaks located at 350 nm and 450 nm, which matches the 4f→5d transition of Ce^3+^. The PL peak of LuAG:Ce^3+^ is located at 512 nm, which is equivalent to the transitions of 5d_1_→^2^F_5/2_ and 5d_1_→^2^F_7/2_. With the increase in the Ce^3+^ concentration, the PL intensity decreases first and then increases in a small range, which may be related to the difference in the crystallinity of ceramics. The SEM micrograph of the surface region of LuAG:0.013Ce^3+^ ceramics are displayed in Figure 1d. The samples have a dense structure, few micropores and defects, and the grain size was almost the same at 10–12 μm. From the EDS mapping in Figure S2, it can be seen that the elements of Lu, Al, O, and Ce are evenly distributed. This result confirmed that high-quality ceramics have been prepared and provides a good foundation for optical information storage.

**Figure 2:**
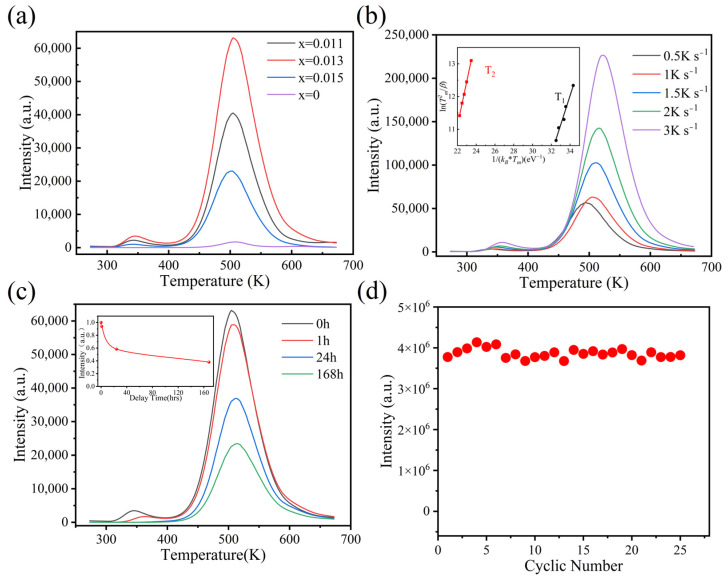
(**a**) TL curves of LuAG:xCe^3+^. (**b**) TL curves with different heating rate of LuAG:0.013Ce^3+^ (Inset: Heating rate plots of LuAG:0.013Ce^3+^). (**c**) TL curves of LuAG:0.013Ce^3+^ for different delay time (Inset: Integral area of TL curves as function of delay time). (**d**) The rewritability test of LuAG:0.013Ce^3+^.

Trap levels are closely related to the information storage properties of ETMs; they are generally analyzed by the thermoluminescence (TL) curve. TL involves the process in which storage optical energy by irradiation with light is re-emitted by heating. As an effective tool to study the trap levels, the TL curve provide lots of information about the traps. The peak position is related to the trap depth, and the peak intensity is related to the number of traps. Therefore, the TL curves of the LuAG:xCe^3+^ materials were measured at 1 K s^−1^ in Figure 2a. There are two different depths of trap (T_1_ and T_2_). Compared with the pure LuAG, the TL intensity of LuAG:0.013Ce^3+^ is significantly improved. The calculation of the depth is as shown in Figure 2b. The specific calculation method is shown in the following formula [35,36]:

(1) βEkBTm2=sexp(−EkBTm)

where *E (eV)*, *β (K/s)*, and *k_B_* are the trap depth, heating rate, and Boltzmann constant; *T_m_* (K) is the temperature corresponding to the maximum vertical axis; and *s* (s^−1^) is the frequency factor. In Figure 2b, By plotting ln($T_{m}^{2}/\beta$) against 1/(*k_B_T_m_*), the trap depth of the TL curve can be determined from the slope of the straight line. When x = 0.013, the trap depths are 1.36 eV and 0.89 eV, respectively, which are larger than those of most typical ETMs [37]. Next, the information retention ability of the sample was tested, which was expressed as the integral area under the TL curves. As shown in Figure 2c, the TL curve of the LuAG:0.013Ce^3+^ materials were tested for different decay times after being irradiated by 254 nm light. With the decay time increasing, the signal intensity attenuates and gradually flattens out. After a delay for 168 h, the sample still has about 40% integrated intensity, and the stability of information storage is slightly lower than that of the YAG: Ce crystal [38]. This indicates that the sample has a relatively stable information storage capacity.

Rewritability is one of the most important properties for electron-trapping materials. Therefore, the rewritability of the LuAG:Ce material was verified. This cycle includes write-in, readout, and erasing. Firstly, UV light is used to excite the ceramic for 1 min; then, the ceramic is heated to 673 K after 30 s, and the data were recorded during heating. Finally, the sample is cooled down to room temperature. The area integral of the TL curve in each cycle is taken as the optical signal intensity, as shown in Figure 2d. Obviously, after 25 cycles, the signal intensity remains basically unchanged, and the size fluctuated around 7%, which was basically the same as the literature results [21]. This is due to the electron capture and de-trapping mechanism. In addition, it is proving that the sample has a higher physical and chemical stability than sulfide and glass phosphors [17,18,24].

### 3.2. Analysis of Trap and Optical Storage Principle

From the analysis of the TL glow curve, we know that there are two traps of different depths in LuAG:Ce. This may be due to the difference in the attraction of the traps to the carriers, which makes the carrier trapped in different positions around the traps. The principle of information storage is based on the optical quantum effect of the trap level on electron capture and release. Therefore, it is necessary to study the transfer process of electrons clearly. In LuAG:Ce, Ce^3+^ is not only the luminescence center [39], but also provides electrons; this is because Ce^3+^ is inclined to lose electrons and become Ce^4+^, due to its special external electron configuration [40,41]. These electrons are captured by traps and stored stably. This explains the significant enhancement of the TL intensity of LuAG with Ce^3+^ doping.

**Figure 3:**
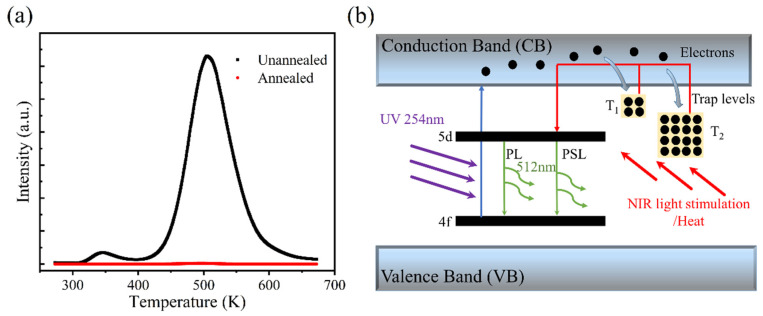
(**a**) TL curves of LuAG:Ce before and after annealing at 1000 °C. (**b**) A diagram of the information storage principle.

For the nature of the trap level, it is generally considered to have oxygen vacancies [42,43], and few Lu_Al,16a_ anti-site defects (ADs) [44]. Herein, the samples were prepared in a high-temperature vacuum environment, which is more likely to generate defects. To study the origin of defects, the TL intensity of the sample was tested after high-temperature annealing in air, and the TL curves are displayed in Figure 3a. Compared with the unannealed sample, the TL intensity of the sample after air annealing almost disappeared; this is because oxygen vacancies are filled by oxygen under the high-temperature and oxygen-rich atmosphere [45,46]. The above results indicate that the trap levels in the material are related to oxygen vacancies.

On the basis of the above experiments, an information storage principle of the LuAG:Ce material was proposed, as shown in Figure 3b. After the ceramic is irradiated, the electrons on the Ce^3+^ ground state 4f are excited to the 5d energy level and continue to the conduction band. Some activated electrons in the conduction band return to the 5d energy level and transition to the 4f ground state with 512 nm. The other electrons can transfer to the conduction band (CB) and are captured by trap levels. Electrons are released from the trap when stimulated by heating or 980 nm and transfer to the Ce^3+^ excited state energy level and ground state, resulting in the green PSL (readout).

### 3.3. Performance of ODS

**Figure 4:**
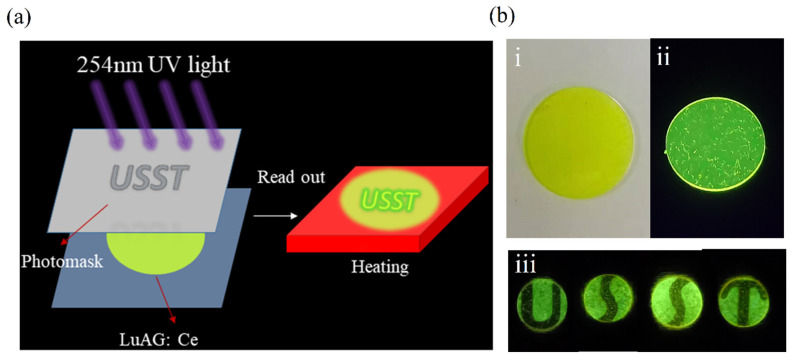
(**a**) Diagram of the optical storage application. (**b**) Photos of LuAG:Ce under (**i**) sunlight; (**ii**) under 254 nm light; (**iii**) the sample was covered with photomask “U”, “S”, “S”, and “T” for 5 min and heated to 250 °C.

An experimental scheme of the optical storage and readout is shown in Figure 4a. The 254 nm UV light is irradiated to the sample through the mask. The electrons in the material are excited and transitioned to the CB, and then captured by the deep trap level and stabilized for a period of time, that is, the letters “USST” are recorded into the storage medium, and the data are read by heating the sample. Figure 4b was photographed in sunlight (i) and 254 nm light (ii). As shown in Figure 4b(iii), LuAG:Ce was covered with a mask, then irradiated with a 254 nm UV lamp for 5 min, and heated to 250 °C; the letters “U, S, S, and T” were read out, and the unirradiated area was black.

**Figure 5:**
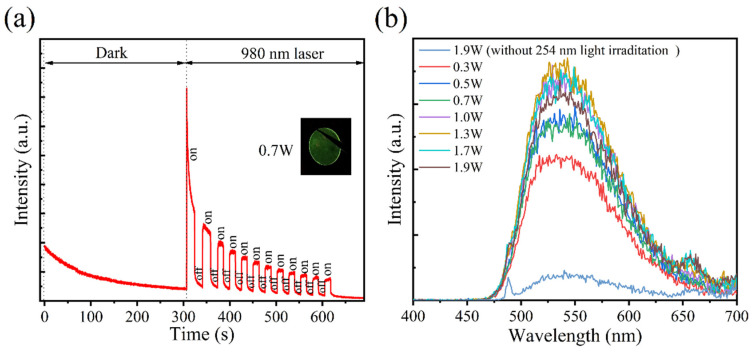
Sample was excited by 254 nm light for 15 min. (**a**) Luminescence decay curve of the sample. (**b**) Photostimulated luminescence (PSL) curves of the ceramic by 980 nm laser in different power settings.

Furthermore, the reading of information by NIR photostimulation (980 nm laser) was demonstrated, as shown in Figure 5a. The luminescence decay curve was recorded at room temperature. The sample produced persistent luminescent (PersL) in the dark due to the shallow traps. After 300 s, we used the 980 nm laser (0.7 W) in a periodically on/off mode to excite the sample and recorded the intensity change. A bright emission from the sample was observed after turning on the 980 nm laser, and the intensity was much higher than the PersL. With the increase in laser irradiation time, the intensity gradually decreases, since the electrons in the trap are gradually released by the 980 nm laser. The PSL emission spectra by using the 980 nm laser in different power modes were tested, and as shown in Figure 5b, the emission peak position is basically consistent with Figure 1c. In addition, with the increase in laser power, the emission intensity first increases and then decreases, which corresponds with the “readout” and “erasing” of the 980 nm laser. The above results show that it is possible to readout information at a high speed by the 980 nm laser, which is of great significance to information storage.

## 4. Conclusions

In conclusion, we prepared Lu_3−x_Al_5_O_12_:xCe (x = 0.011, 0.013 and 0.015) fluorescent ceramics by the vacuum sintering technique, and its crystal structure, trap level, and optical storage characteristics were systematically studied. The TL intensity is significantly increased by the selective introduction of Ce^3+^; it acts as a luminescence center. The lutetium aluminum garnet-structured ceramics ensure good cycle stability (25 times). The nature of the trap was discussed, and is it believed that the oxygen vacancies generated by the vacuum sintering environment capture electrons and lead to PSL and TL. Finally, the thermally stimulated readout and PSL properties of the sample were verified, demonstrating the potential of LuAG:Ce in field of optical storage. Currently, LuAG:Ce’s short information storage time and heating-based readout method limit its ODS application. With these results, we hope to promote the development of new storage technology by optimizing material design and combining advanced optical technologies.

## Data Availability

The original contributions presented in this study are included in the article/Supplementary Materials. Further inquiries can be directed to the corresponding author.

## Supplementary Materials

The following supporting information can be downloaded at: https://www.mdpi.com/article/10.3390/ma18010063/s1, Figure S1: Flowchart of the preparation of LuAG:Ce ceramics; Figure S2: Flowchart of the preparation of LuAG:Ce ceramics.
