# Supplementary material for: Lu3Al5O12:Ce3+ Fluorescent Ceramic with Deep Traps: Thermoluminescence and Photostimulable Luminescence Properties
Source: Materials (Basel). 2024 Dec 27;18(1):63. doi: 10.3390/ma18010063 (PMC11721196; doi:10.3390/ma18010063)
Supplement: Supplementary file 1 [file materials-18-00063-s001.zip › materials-3312547-supplementary.pdf]

$Lu_3Al_5O_{12}:Ce^{3+}$ Fluorescent Ceramic with Deep Traps: Thermoluminescence and Photostimulable Luminescence Properties

Junwei Zhang [1], Miao Zhao [2], Qiao Hu [2], Renjie Jiang [2], Hao Ruan [2,*] and Hui Lin [1]

[1] Engineering Research Center of Optical Instrument and System, Ministry of Education and Shanghai Key Lab of Modern Optical System, University of Shanghai for Science and Technology, No. 516 Jungong Road, Shanghai 200093, China; junweizhang@siom.ac.cn (J.Z.); linh8112@163.com (H.L.)
[2] Aerospace Laser Technology and System Department, Wangzhijiang Innovation Center for Laser, Shanghai Institute of Optics and Fine Mechanics, Chinese Academy of Sciences, Shanghai 201800, China; miaozhao@siom.ac.cn (M.Z.); huqiao@siom.ac.cn (Q.H.); jiangrj@shanghaitech.edu.cn (R.J.)
* Correspondence: ruanhao@siom.ac.cn

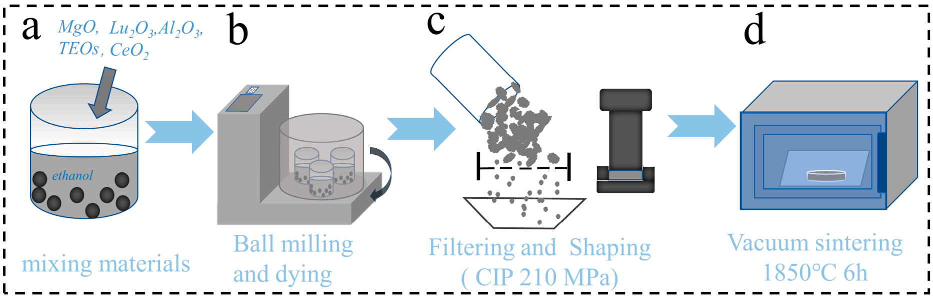

Figure S1. Flowchart of the preparation of LuAG:Ce ceramics.

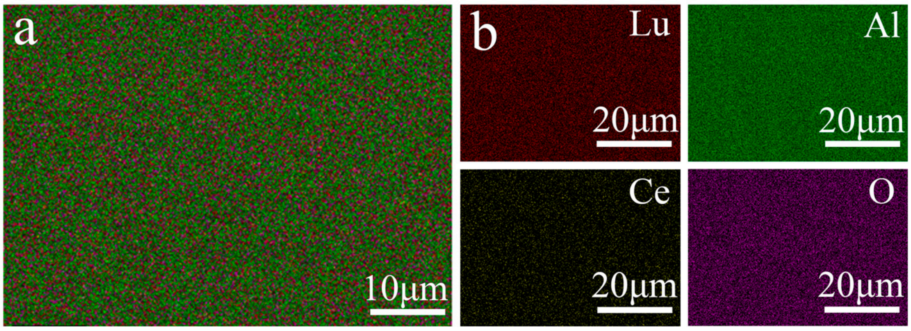

Figure S2. EDS mapping of the LuAG:0.013Ce^{3+}.
